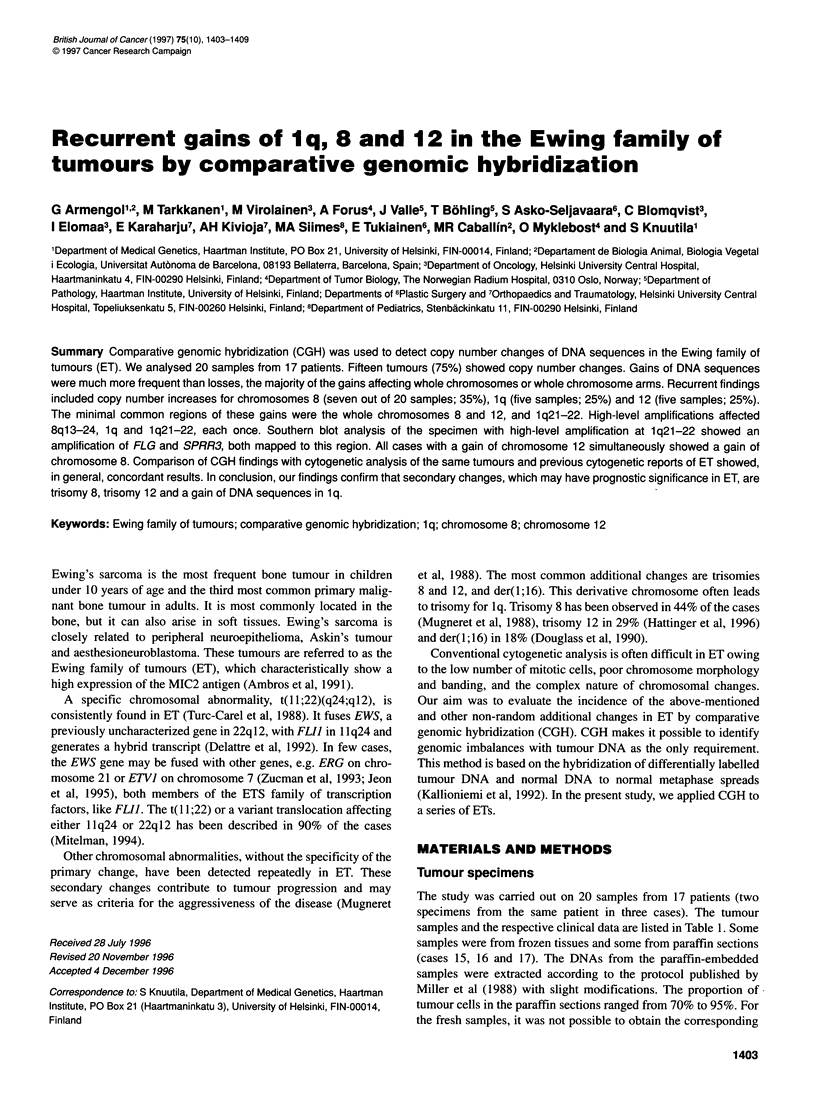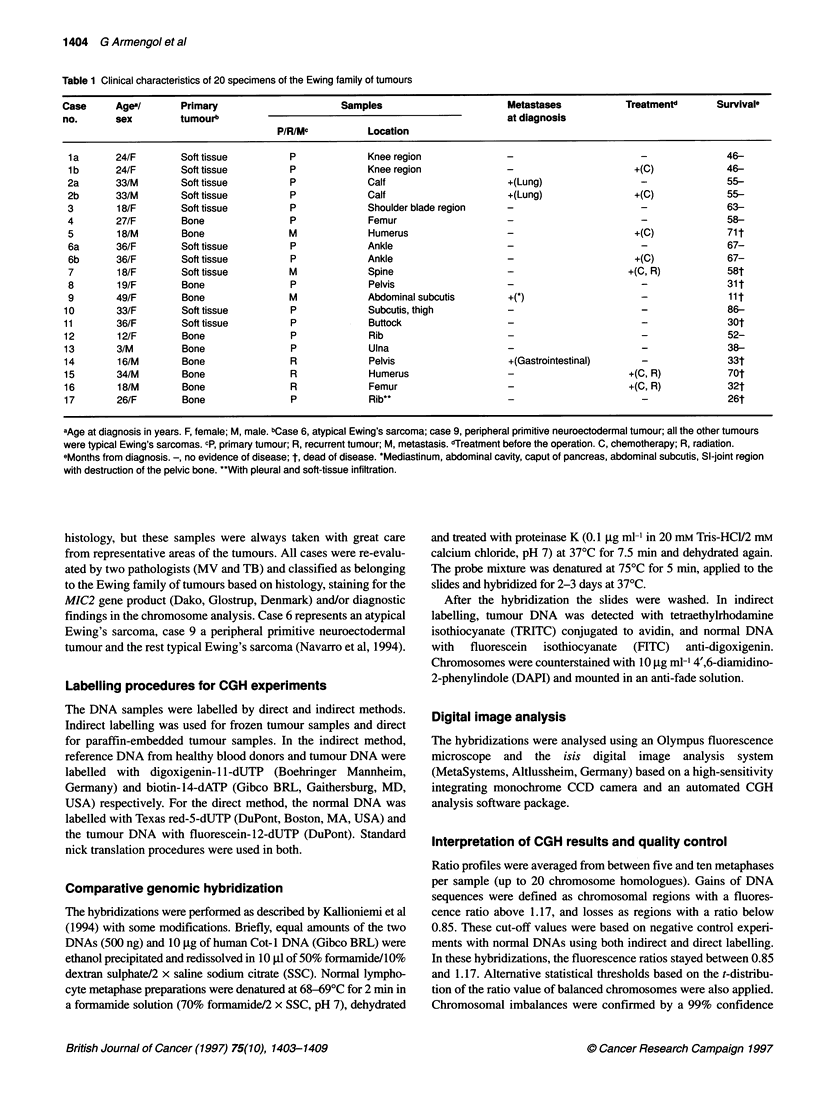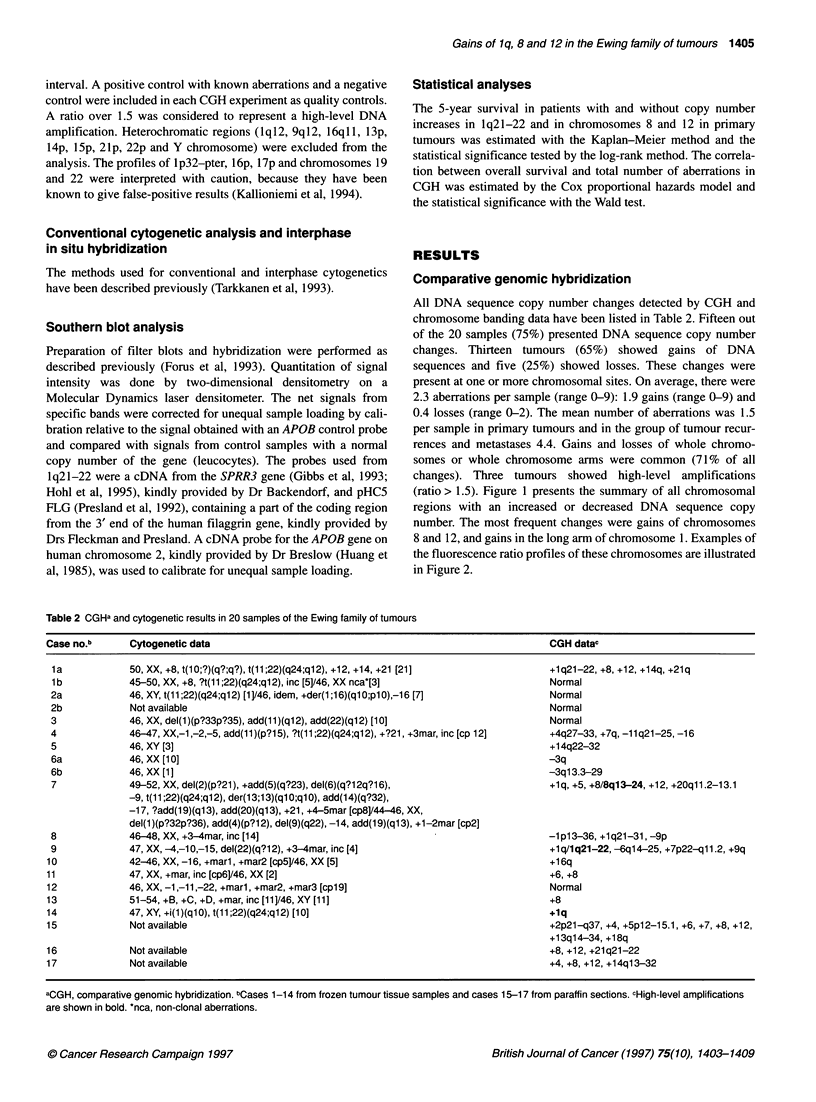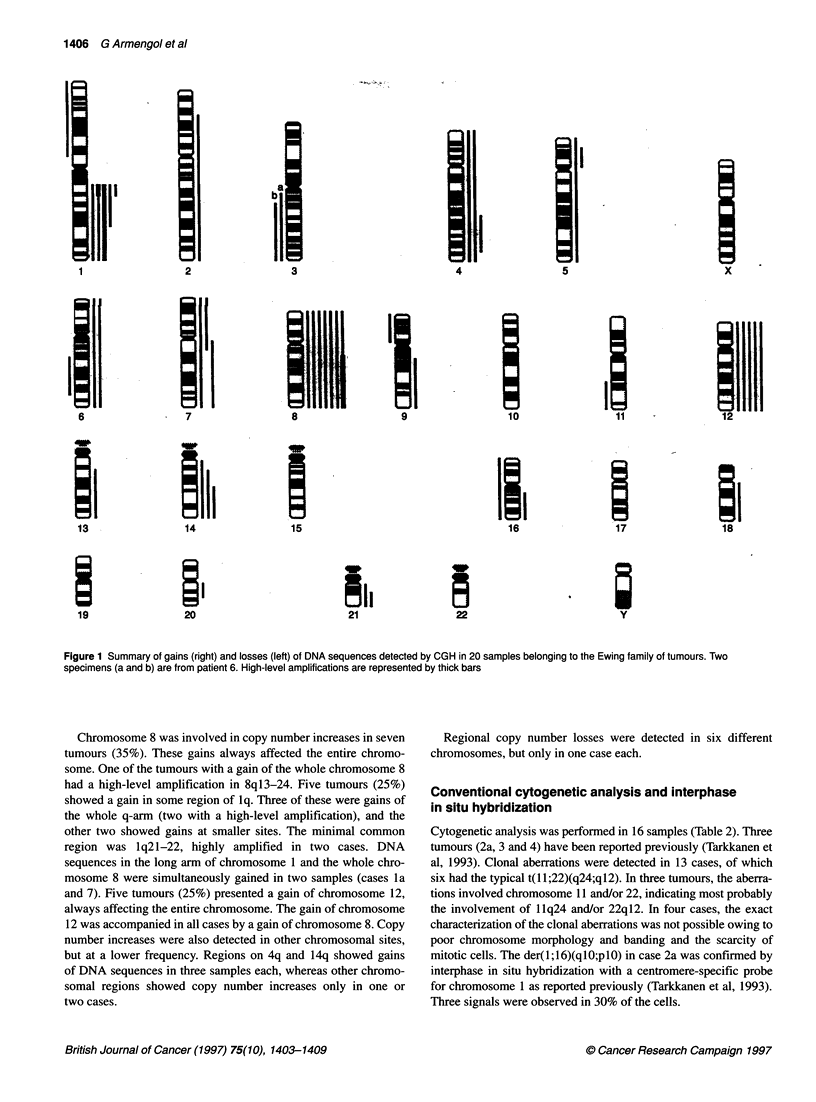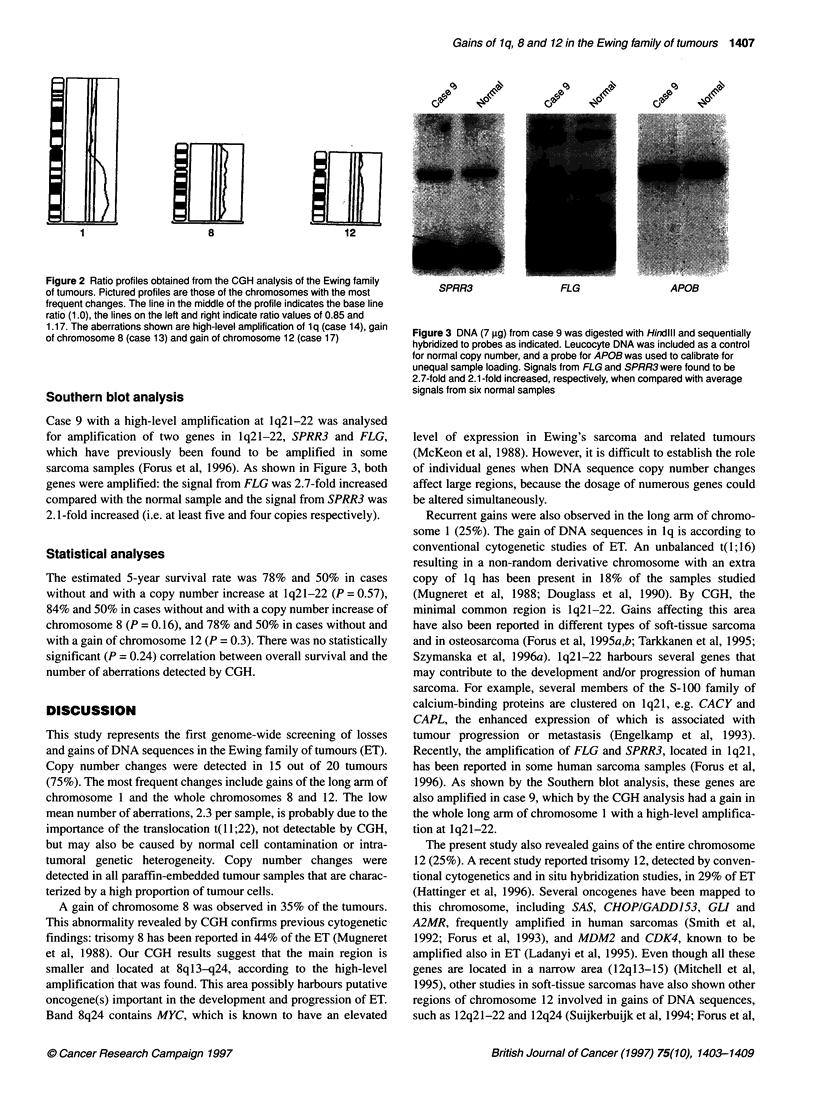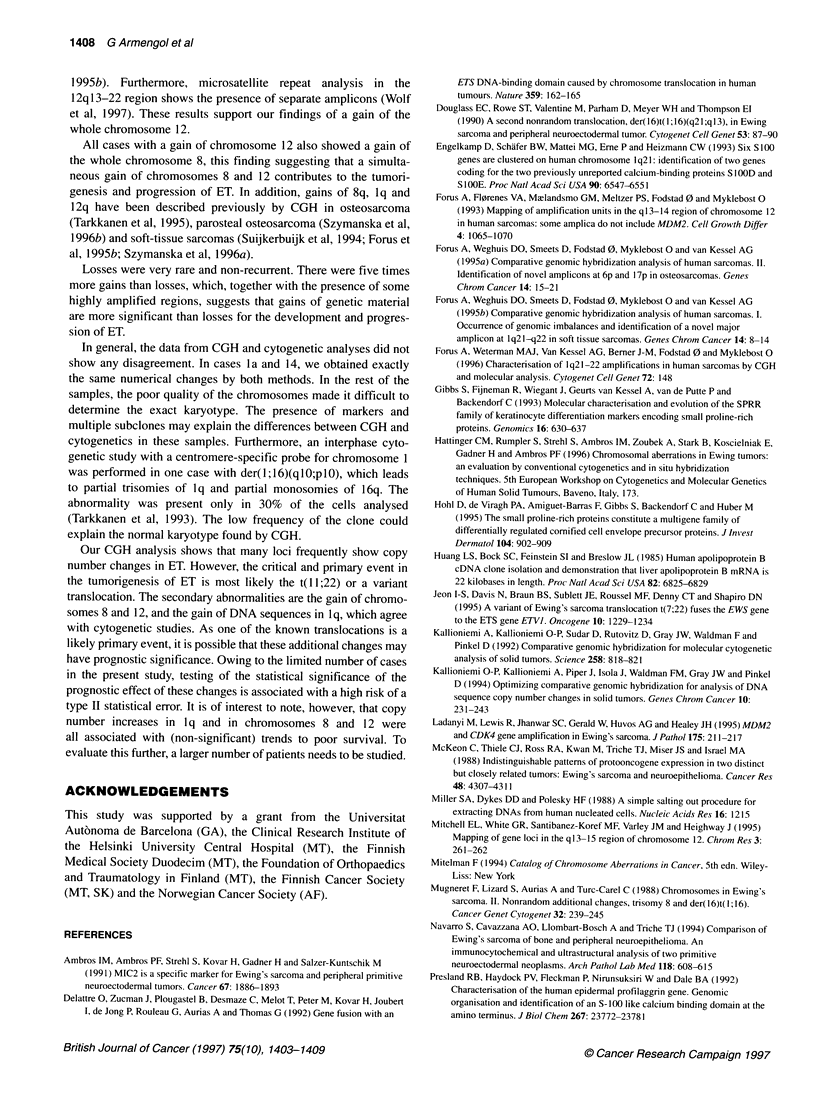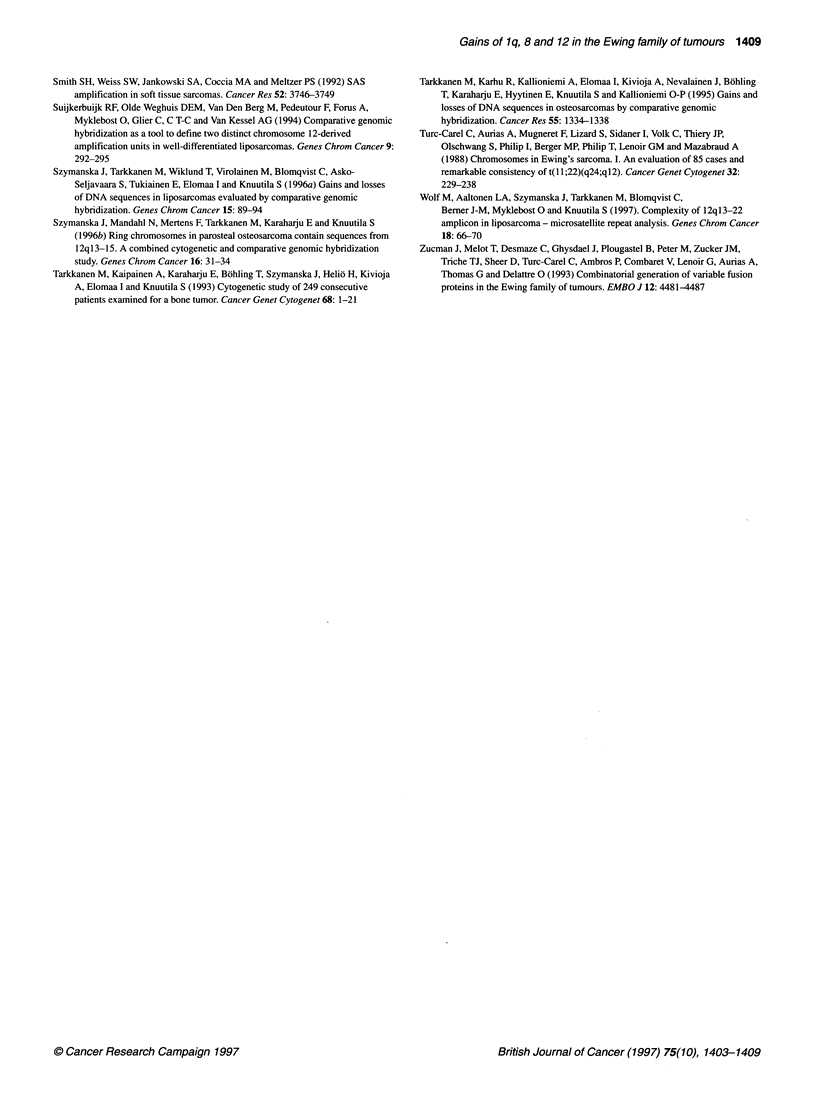# Recurrent gains of 1q, 8 and 12 in the Ewing family of tumours by comparative genomic hybridization.

**DOI:** 10.1038/bjc.1997.242

**Published:** 1997

**Authors:** G. Armengol, M. Tarkkanen, M. Virolainen, A. Forus, J. Valle, T. BÃ¶hling, S. Asko-Seljavaara, C. Blomqvist, I. Elomaa, E. Karaharju, A. H. Kivioja, M. A. Siimes, E. Tukiainen, M. R. CaballÃ­n, O. Myklebost, S. Knuutila

**Affiliations:** Department of Medical Genetics, Haartman Institute, University of Helsinki, Finland.

## Abstract

Comparative genomic hybridization (CGH) was used to detect copy number changes of DNA sequences in the Ewing family of tumours (ET). We analysed 20 samples from 17 patients. Fifteen tumours (75%) showed copy number changes. Gains of DNA sequences were much more frequent than losses, the majority of the gains affecting whole chromosomes or whole chromosome arms. Recurrent findings included copy number increases for chromosomes 8 (seven out of 20 samples; 35%), 1q (five samples; 25%) and 12 (five samples; 25%). The minimal common regions of these gains were the whole chromosomes 8 and 12, and 1q21-22. High-level amplifications affected 8q13-24, 1q and 1q21-22, each once. Southern blot analysis of the specimen with high-level amplification at 1q21-22 showed an amplification of FLG and SPRR3, both mapped to this region. All cases with a gain of chromosome 12 simultaneously showed a gain of chromosome 8. Comparison of CGH findings with cytogenetic analysis of the same tumours and previous cytogenetic reports of ET showed, in general, concordant results. In conclusion, our findings confirm that secondary changes, which may have prognostic significance in ET, are trisomy 8, trisomy 12 and a gain of DNA sequences in 1q.


British Journal of Cancer (1997) 75(10), 1403–1409
© 1997 Cancer Research Campaign

### Recurrent gains of 1q, 8 and 12 in the Ewing family of tumours by comparative genomic hybridization

**G Armengol[1,2], M Tarkkanen[1], M Virolainen[3], A Forus[4], J Valle[5], T Böhling[5], S Asko-Seljavaara[6], C Blomqvist[3], I Elomaa[3], E Karaharju[7], AH Kivioja[7], MA Siimes[8], E Tukiainen[6], MR Caballín[2], O Myklebost[4] and S Knuutila[1]**

[1]Department of Medical Genetics, Haartman Institute, PO Box 21, University of Helsinki, FIN-00014, Finland; [2]Departament de Biologia Animal, Biologia Vegetal i Ecologia, Universitat Autònoma de Barcelona, 08193 Bellaterra, Barcelona, Spain; [3]Department of Oncology, Helsinki University Central Hospital, Haartmaninkatu 4, FIN-00290 Helsinki, Finland; [4]Department of Tumor Biology, The Norwegian Radium Hospital, 0310 Oslo, Norway; [5]Department of Pathology, Haartman Institute, University of Helsinki, Finland; Departments of [6]Plastic Surgery and [7]Orthopaedics and Traumatology, Helsinki University Central Hospital, Topeliuksenkatu 5, FIN-00260 Helsinki, Finland; [8]Department of Pediatrics, Stenbäckinkatu 11, FIN-00290 Helsinki, Finland

**Summary** Comparative genomic hybridization (CGH) was used to detect copy number changes of DNA sequences in the Ewing family of tumours (ET). We analysed 20 samples from 17 patients. Fifteen tumours (75%) showed copy number changes. Gains of DNA sequences were much more frequent than losses, the majority of the gains affecting whole chromosomes or whole chromosome arms. Recurrent findings included copy number increases for chromosomes 8 (seven out of 20 samples; 35%), 1q (five samples; 25%) and 12 (five samples; 25%). The minimal common regions of these gains were the whole chromosomes 8 and 12, and 1q21–22. High-level amplifications affected 8q13–24, 1q and 1q21–22, each once. Southern blot analysis of the specimen with high-level amplification at 1q21–22 showed an amplification of *FLG* and *SPRR3*, both mapped to this region. All cases with a gain of chromosome 12 simultaneously showed a gain of chromosome 8. Comparison of CGH findings with cytogenetic analysis of the same tumours and previous cytogenetic reports of ET showed, in general, concordant results. In conclusion, our findings confirm that secondary changes, which may have prognostic significance in ET, are trisomy 8, trisomy 12 and a gain of DNA sequences in 1q.

**Keywords:** Ewing family of tumours; comparative genomic hybridization; 1q; chromosome 8; chromosome 12

Ewing's sarcoma is the most frequent bone tumour in children under 10 years of age and the third most common primary malignant bone tumour in adults. It is most commonly located in the bone, but it can also arise in soft tissues. Ewing's sarcoma is closely related to peripheral neuroepithelioma, Askin's tumour and aesthesioneuroblastoma. These tumours are referred to as the Ewing family of tumours (ET), which characteristically show a high expression of the MIC2 antigen (Ambros et al, 1991).

A specific chromosomal abnormality, t(11;22)(q24;q12), is consistently found in ET (Turc-Carel et al, 1988). It fuses *EWS*, a previously uncharacterized gene in 22q12, with *FLI1* in 11q24 and generates a hybrid transcript (Delattre et al, 1992). In few cases, the *EWS* gene may be fused with other genes, e.g. *ERG* on chromosome 21 or *ETV1* on chromosome 7 (Zucman et al, 1993; Jeon et al, 1995), both members of the ETS family of transcription factors, like *FLI1*. The t(11;22) or a variant translocation affecting either 11q24 or 22q12 has been described in 90% of the cases (Mitelman, 1994).

Other chromosomal abnormalities, without the specificity of the primary change, have been detected repeatedly in ET. These secondary changes contribute to tumour progression and may serve as criteria for the aggressiveness of the disease (Mugneret et al, 1988). The most common additional changes are trisomies 8 and 12, and der(1;16). This derivative chromosome often leads to trisomy for 1q. Trisomy 8 has been observed in 44% of the cases (Mugneret et al, 1988), trisomy 12 in 29% (Hattinger et al, 1996) and der(1;16) in 18% (Douglass et al, 1990).

Conventional cytogenetic analysis is often difficult in ET owing to the low number of mitotic cells, poor chromosome morphology and banding, and the complex nature of chromosomal changes. Our aim was to evaluate the incidence of the above-mentioned and other non-random additional changes in ET by comparative genomic hybridization (CGH). CGH makes it possible to identify genomic imbalances with tumour DNA as the only requirement. This method is based on the hybridization of differentially labelled tumour DNA and normal DNA to normal metaphase spreads (Kallioniemi et al, 1992). In the present study, we applied CGH to a series of ETs.

### MATERIALS AND METHODS

### Tumour specimens

The study was carried out on 20 samples from 17 patients (two specimens from the same patient in three cases). The tumour samples and the respective clinical data are listed in Table 1. Some samples were from frozen tissues and some from paraffin sections (cases 15, 16 and 17). The DNAs from the paraffin-embedded samples were extracted according to the protocol published by Miller et al (1988) with slight modifications. The proportion of tumour cells in the paraffin sections ranged from 70% to 95%. For the fresh samples, it was not possible to obtain the corresponding

*Received 28 July 1996*
*Revised 20 November 1996*
*Accepted 4 December 1996*

*Correspondence to:* S Knuutila, Department of Medical Genetics, Haartman Institute, PO Box 21 (Haartmaninkatu 3), University of Helsinki, FIN-00014, Finland

**Table 1** Clinical characteristics of 20 specimens of the Ewing family of tumours

| Case no. | Age[a]/ sex | Primary tumour[b] | Samples | | Metastases at diagnosis | Treatment[d] | Survival[e] |
|---|---|---|---|---|---|---|---|
| | | | P/R/M[c] | Location | | | |
| 1a | 24/F | Soft tissue | P | Knee region | – | – | 46– |
| 1b | 24/F | Soft tissue | P | Knee region | – | +(C) | 46– |
| 2a | 33/M | Soft tissue | P | Calf | +(Lung) | – | 55– |
| 2b | 33/M | Soft tissue | P | Calf | +(Lung) | +(C) | 55– |
| 3 | 18/F | Soft tissue | P | Shoulder blade region | – | – | 63– |
| 4 | 27/F | Bone | P | Femur | – | – | 58– |
| 5 | 18/M | Bone | M | Humerus | – | +(C) | 71† |
| 6a | 36/F | Soft tissue | P | Ankle | – | – | 67– |
| 6b | 36/F | Soft tissue | P | Ankle | – | +(C) | 67– |
| 7 | 18/F | Soft tissue | M | Spine | – | +(C, R) | 58† |
| 8 | 19/F | Bone | P | Pelvis | – | – | 31† |
| 9 | 49/F | Bone | M | Abdominal subcutis | +(*) | – | 11† |
| 10 | 33/F | Soft tissue | P | Subcutis, thigh | – | – | 86– |
| 11 | 36/F | Soft tissue | P | Buttock | – | – | 30† |
| 12 | 12/F | Bone | P | Rib | – | – | 52– |
| 13 | 3/M | Bone | P | Ulna | – | – | 38– |
| 14 | 16/M | Bone | R | Pelvis | +(Gastrointestinal) | – | 33† |
| 15 | 34/M | Bone | R | Humerus | – | +(C, R) | 70† |
| 16 | 18/M | Bone | R | Femur | – | +(C, R) | 32† |
| 17 | 26/F | Bone | P | Rib** | – | – | 26† |

[a]Age at diagnosis in years. F, female; M, male. [b]Case 6, atypical Ewing's sarcoma; case 9, peripheral primitive neuroectodermal tumour; all the other tumours were typical Ewing's sarcomas. [c]P, primary tumour; R, recurrent tumour; M, metastasis. [d]Treatment before the operation. C, chemotherapy; R, radiation. [e]Months from diagnosis. –, no evidence of disease; †, dead of disease. *Mediastinum, abdominal cavity, caput of pancreas, abdominal subcutis, SI-joint region with destruction of the pelvic bone. **With pleural and soft-tissue infiltration.

histology, but these samples were always taken with great care from representative areas of the tumours. All cases were re-evaluated by two pathologists (MV and TB) and classified as belonging to the Ewing family of tumours based on histology, staining for the *MIC2* gene product (Dako, Glostrup, Denmark) and/or diagnostic findings in the chromosome analysis. Case 6 represents an atypical Ewing's sarcoma, case 9 a peripheral primitive neuroectodermal tumour and the rest typical Ewing's sarcoma (Navarro et al, 1994).

### Labelling procedures for CGH experiments

The DNA samples were labelled by direct and indirect methods. Indirect labelling was used for frozen tumour samples and direct for paraffin-embedded tumour samples. In the indirect method, reference DNA from healthy blood donors and tumour DNA were labelled with digoxigenin-11-dUTP (Boehringer Mannheim, Germany) and biotin-14-dATP (Gibco BRL, Gaithersburg, MD, USA) respectively. For the direct method, the normal DNA was labelled with Texas red-5-dUTP (DuPont, Boston, MA, USA) and the tumour DNA with fluorescein-12-dUTP (DuPont). Standard nick translation procedures were used in both.

### Comparative genomic hybridization

The hybridizations were performed as described by Kallioniemi et al (1994) with some modifications. Briefly, equal amounts of the two DNAs (500 ng) and 10 μg of human Cot-1 DNA (Gibco BRL) were ethanol precipitated and redissolved in 10 μl of 50% formamide/10% dextran sulphate/2 × saline sodium citrate (SSC). Normal lymphocyte metaphase preparations were denatured at 68–69°C for 2 min in a formamide solution (70% formamide/2 × SSC, pH 7), dehydrated and treated with proteinase K (0.1 μg $ml^{-1}$ in 20 mM Tris-HCl/2 mM calcium chloride, pH 7) at 37°C for 7.5 min and dehydrated again. The probe mixture was denatured at 75°C for 5 min, applied to the slides and hybridized for 2–3 days at 37°C.

After the hybridization the slides were washed. In indirect labelling, tumour DNA was detected with tetraethylrhodamine isothiocyanate (TRITC) conjugated to avidin, and normal DNA with fluorescein isothiocyanate (FITC) anti-digoxigenin. Chromosomes were counterstained with 10 μg $ml^{-1}$ 4′,6-diamidino-2-phenylindole (DAPI) and mounted in an anti-fade solution.

### Digital image analysis

The hybridizations were analysed using an Olympus fluorescence microscope and the *isis* digital image analysis system (MetaSystems, Altlussheim, Germany) based on a high-sensitivity integrating monochrome CCD camera and an automated CGH analysis software package.

### Interpretation of CGH results and quality control

Ratio profiles were averaged from between five and ten metaphases per sample (up to 20 chromosome homologues). Gains of DNA sequences were defined as chromosomal regions with a fluorescence ratio above 1.17, and losses as regions with a ratio below 0.85. These cut-off values were based on negative control experiments with normal DNAs using both indirect and direct labelling. In these hybridizations, the fluorescence ratios stayed between 0.85 and 1.17. Alternative statistical thresholds based on the *t*-distribution of the ratio value of balanced chromosomes were also applied. Chromosomal imbalances were confirmed by a 99% confidence

 © Cancer Research Campaign 1997

interval. A positive control with known aberrations and a negative control were included in each CGH experiment as quality controls. A ratio over 1.5 was considered to represent a high-level DNA amplification. Heterochromatic regions (1q12, 9q12, 16q11, 13p, 14p, 15p, 21p, 22p and Y chromosome) were excluded from the analysis. The profiles of 1p32–pter, 16p, 17p and chromosomes 19 and 22 were interpreted with caution, because they have been known to give false-positive results (Kallioniemi et al, 1994).

### Conventional cytogenetic analysis and interphase in situ hybridization

The methods used for conventional and interphase cytogenetics have been described previously (Tarkkanen et al, 1993).

### Southern blot analysis

Preparation of filter blots and hybridization were performed as described previously (Forus et al, 1993). Quantitation of signal intensity was done by two-dimensional densitometry on a Molecular Dynamics laser densitometer. The net signals from specific bands were corrected for unequal sample loading by calibration relative to the signal obtained with an *APOB* control probe and compared with signals from control samples with a normal copy number of the gene (leucocytes). The probes used from 1q21–22 were a cDNA from the *SPRR3* gene (Gibbs et al, 1993; Hohl et al, 1995), kindly provided by Dr Backendorf, and pHC5 FLG (Presland et al, 1992), containing a part of the coding region from the 3′ end of the human filaggrin gene, kindly provided by Drs Fleckman and Presland. A cDNA probe for the *APOB* gene on human chromosome 2, kindly provided by Dr Breslow (Huang et al, 1985), was used to calibrate for unequal sample loading.

### Statistical analyses

The 5-year survival in patients with and without copy number increases in 1q21–22 and in chromosomes 8 and 12 in primary tumours was estimated with the Kaplan–Meier method and the statistical significance tested by the log-rank method. The correlation between overall survival and total number of aberrations in CGH was estimated by the Cox proportional hazards model and the statistical significance with the Wald test.

### RESULTS

### Comparative genomic hybridization

All DNA sequence copy number changes detected by CGH and chromosome banding data have been listed in Table 2. Fifteen out of the 20 samples (75%) presented DNA sequence copy number changes. Thirteen tumours (65%) showed gains of DNA sequences and five (25%) showed losses. These changes were present at one or more chromosomal sites. On average, there were 2.3 aberrations per sample (range 0–9): 1.9 gains (range 0–9) and 0.4 losses (range 0–2). The mean number of aberrations was 1.5 per sample in primary tumours and in the group of tumour recurrences and metastases 4.4. Gains and losses of whole chromosomes or whole chromosome arms were common (71% of all changes). Three tumours showed high-level amplifications (ratio > 1.5). Figure 1 presents the summary of all chromosomal regions with an increased or decreased DNA sequence copy number. The most frequent changes were gains of chromosomes 8 and 12, and gains in the long arm of chromosome 1. Examples of the fluorescence ratio profiles of these chromosomes are illustrated in Figure 2.

**Table 2** CGH[a] and cytogenetic results in 20 samples of the Ewing family of tumours

| Case no.[b] | Cytogenetic data | CGH data[c] |
|---|---|---|
| 1a | 50, XX, +8, t(10;?)(q?;q?), t(11;22)(q24;q12), +12, +14, +21 [21] | +1q21–22, +8, +12, +14q, +21q |
| 1b | 45–50, XX, +8, ?t(11;22)(q24;q12), inc [5]/46, XX nca*[3] | Normal |
| 2a | 46, XY, t(11;22)(q24;q12) [1]/46, idem, +der(1;16)(q10;p10),–16 [7] | Normal |
| 2b | Not available | Normal |
| 3 | 46, XX, del(1)(p?33p?35), add(11)(q12), add(22)(q12) [10] | Normal |
| 4 | 46–47, XX,–1,–2,–5, add(11)(p?15), ?t(11;22)(q24;q12), +?21, +3mar, inc [cp 12] | +4q27–33, +7q, –11q21–25, –16 |
| 5 | 46, XY [3] | +14q22–32 |
| 6a | 46, XX [10] | –3q |
| 6b | 46, XX [1] | –3q13.3–29 |
| 7 | 49–52, XX, del(2)(p?21), +add(5)(q?23), del(6)(q?12q?16), –9, t(11;22)(q24;q12), der(13;13)(q10;q10), add(14)(q?32), –17, ?add(19)(q13), add(20)(q13), +21, +4–5mar [cp8]/44–46, XX, del(1)(p?32p?36), add(4)(p?12), del(9)(q22), –14, add(19)(q13), +1–2mar [cp2] | +1q, +5, +8/**8q13–24**, +12, +20q11.2–13.1 |
| 8 | 46–48, XX, +3–4mar, inc [14] | –1p13–36, +1q21–31, –9p |
| 9 | 47, XX, –4,–10,–15, del(22)(q?12), +3–4mar, inc [4] | +1q/**1q21–22**, –6q14–25, +7p22–q11.2, +9q |
| 10 | 42–46, XX, –16, +mar1, +mar2 [cp5]/46, XX [5] | +16q |
| 11 | 47, XX, +mar, inc [cp6]/46, XX [2] | +6, +8 |
| 12 | 46, XX, –1,–11,–22, +mar1, +mar2, +mar3 [cp19] | Normal |
| 13 | 51–54, +B, +C, +D, +mar, inc [11]/46, XY [11] | +8 |
| 14 | 47, XY, +i(1)(q10), t(11;22)(q24;q12) [10] | **+1q** |
| 15 | Not available | +2p21–q37, +4, +5p12–15.1, +6, +7, +8, +12, +13q14–34, +18q |
| 16 | Not available | +8, +12, +21q21–22 |
| 17 | Not available | +4, +8, +12, +14q13–32 |

[a]CGH, comparative genomic hybridization. [b]Cases 1–14 from frozen tumour tissue samples and cases 15–17 from paraffin sections. [c]High-level amplifications are shown in bold. *nca, non-clonal aberrations.

© Cancer Research Campaign 1997

**Figure 1** Summary of gains (right) and losses (left) of DNA sequences detected by CGH in 20 samples belonging to the Ewing family of tumours. Two specimens (a and b) are from patient 6. High-level amplifications are represented by thick bars

Chromosome 8 was involved in copy number increases in seven tumours (35%). These gains always affected the entire chromosome. One of the tumours with a gain of the whole chromosome 8 had a high-level amplification in 8q13–24. Five tumours (25%) showed a gain in some region of 1q. Three of these were gains of the whole q-arm (two with a high-level amplification), and the other two showed gains at smaller sites. The minimal common region was 1q21–22, highly amplified in two cases. DNA sequences in the long arm of chromosome 1 and the whole chromosome 8 were simultaneously gained in two samples (cases 1a and 7). Five tumours (25%) presented a gain of chromosome 12, always affecting the entire chromosome. The gain of chromosome 12 was accompanied in all cases by a gain of chromosome 8. Copy number increases were also detected in other chromosomal sites, but at a lower frequency. Regions on 4q and 14q showed gains of DNA sequences in three samples each, whereas other chromosomal regions showed copy number increases only in one or two cases.

Regional copy number losses were detected in six different chromosomes, but only in one case each.

### Conventional cytogenetic analysis and interphase in situ hybridization

Cytogenetic analysis was performed in 16 samples (Table 2). Three tumours (2a, 3 and 4) have been reported previously (Tarkkanen et al, 1993). Clonal aberrations were detected in 13 cases, of which six had the typical t(11;22)(q24;q12). In three tumours, the aberrations involved chromosome 11 and/or 22, indicating most probably the involvement of 11q24 and/or 22q12. In four cases, the exact characterization of the clonal aberrations was not possible owing to poor chromosome morphology and banding and the scarcity of mitotic cells. The der(1;16)(q10;p10) in case 2a was confirmed by interphase in situ hybridization with a centromere-specific probe for chromosome 1 as reported previously (Tarkkanen et al, 1993). Three signals were observed in 30% of the cells.

© Cancer Research Campaign 1997

**Figure 2** Ratio profiles obtained from the CGH analysis of the Ewing family of tumours. Pictured profiles are those of the chromosomes with the most frequent changes. The line in the middle of the profile indicates the base line ratio (1.0), the lines on the left and right indicate ratio values of 0.85 and 1.17. The aberrations shown are high-level amplification of 1q (case 14), gain of chromosome 8 (case 13) and gain of chromosome 12 (case 17)

**Figure 3** DNA (7 μg) from case 9 was digested with *Hin*dIII and sequentially hybridized to probes as indicated. Leucocyte DNA was included as a control for normal copy number, and a probe for *APOB* was used to calibrate for unequal sample loading. Signals from *FLG* and *SPRR3* were found to be 2.7-fold and 2.1-fold increased, respectively, when compared with average signals from six normal samples

### Southern blot analysis

Case 9 with a high-level amplification at 1q21–22 was analysed for amplification of two genes in 1q21–22, *SPRR3* and *FLG*, which have previously been found to be amplified in some sarcoma samples (Forus et al, 1996). As shown in Figure 3, both genes were amplified: the signal from *FLG* was 2.7-fold increased compared with the normal sample and the signal from *SPRR3* was 2.1-fold increased (i.e. at least five and four copies respectively).

### Statistical analyses

The estimated 5-year survival rate was 78% and 50% in cases without and with a copy number increase at 1q21–22 ($P = 0.57$), 84% and 50% in cases without and with a copy number increase of chromosome 8 ($P = 0.16$), and 78% and 50% in cases without and with a gain of chromosome 12 ($P = 0.3$). There was no statistically significant ($P = 0.24$) correlation between overall survival and the number of aberrations detected by CGH.

### DISCUSSION

This study represents the first genome-wide screening of losses and gains of DNA sequences in the Ewing family of tumours (ET). Copy number changes were detected in 15 out of 20 tumours (75%). The most frequent changes include gains of the long arm of chromosome 1 and the whole chromosomes 8 and 12. The low mean number of aberrations, 2.3 per sample, is probably due to the importance of the translocation t(11;22), not detectable by CGH, but may also be caused by normal cell contamination or intratumoral genetic heterogeneity. Copy number changes were detected in all paraffin-embedded tumour samples that are characterized by a high proportion of tumour cells.

A gain of chromosome 8 was observed in 35% of the tumours. This abnormality revealed by CGH confirms previous cytogenetic findings: trisomy 8 has been reported in 44% of the ET (Mugneret et al, 1988). Our CGH results suggest that the main region is smaller and located at 8q13–q24, according to the high-level amplification that was found. This area possibly harbours putative oncogene(s) important in the development and progression of ET. Band 8q24 contains *MYC*, which is known to have an elevated level of expression in Ewing's sarcoma and related tumours (McKeon et al, 1988). However, it is difficult to establish the role of individual genes when DNA sequence copy number changes affect large regions, because the dosage of numerous genes could be altered simultaneously.

Recurrent gains were also observed in the long arm of chromosome 1 (25%). The gain of DNA sequences in 1q is according to conventional cytogenetic studies of ET. An unbalanced t(1;16) resulting in a non-random derivative chromosome with an extra copy of 1q has been present in 18% of the samples studied (Mugneret et al, 1988; Douglass et al, 1990). By CGH, the minimal common region is 1q21–22. Gains affecting this area have also been reported in different types of soft-tissue sarcoma and in osteosarcoma (Forus et al, 1995*a*,*b*; Tarkkanen et al, 1995; Szymanska et al, 1996*a*). 1q21–22 harbours several genes that may contribute to the development and/or progression of human sarcoma. For example, several members of the S-100 family of calcium-binding proteins are clustered on 1q21, e.g. *CACY* and *CAPL*, the enhanced expression of which is associated with tumour progression or metastasis (Engelkamp et al, 1993). Recently, the amplification of *FLG* and *SPRR3*, located in 1q21, has been reported in some human sarcoma samples (Forus et al, 1996). As shown by the Southern blot analysis, these genes are also amplified in case 9, which by the CGH analysis had a gain in the whole long arm of chromosome 1 with a high-level amplification at 1q21–22.

The present study also revealed gains of the entire chromosome 12 (25%). A recent study reported trisomy 12, detected by conventional cytogenetics and in situ hybridization studies, in 29% of ET (Hattinger et al, 1996). Several oncogenes have been mapped to this chromosome, including *SAS*, *CHOP/GADD153*, *GLI* and *A2MR*, frequently amplified in human sarcomas (Smith et al, 1992; Forus et al, 1993), and *MDM2* and *CDK4*, known to be amplified also in ET (Ladanyi et al, 1995). Even though all these genes are located in a narrow area (12q13–15) (Mitchell et al, 1995), other studies in soft-tissue sarcomas have also shown other regions of chromosome 12 involved in gains of DNA sequences, such as 12q21–22 and 12q24 (Suijkerbuijk et al, 1994; Forus et al,

© Cancer Research Campaign 1997 

1995*b*). Furthermore, microsatellite repeat analysis in the 12q13–22 region shows the presence of separate amplicons (Wolf et al, 1997). These results support our findings of a gain of the whole chromosome 12.

All cases with a gain of chromosome 12 also showed a gain of the whole chromosome 8, this finding suggesting that a simultaneous gain of chromosomes 8 and 12 contributes to the tumorigenesis and progression of ET. In addition, gains of 8q, 1q and 12q have been described previously by CGH in osteosarcoma (Tarkkanen et al, 1995), parosteal osteosarcoma (Szymanska et al, 1996*b*) and soft-tissue sarcomas (Suijkerbuijk et al, 1994; Forus et al, 1995*b*; Szymanska et al, 1996*a*).

Losses were very rare and non-recurrent. There were five times more gains than losses, which, together with the presence of some highly amplified regions, suggests that gains of genetic material are more significant than losses for the development and progression of ET.

In general, the data from CGH and cytogenetic analyses did not show any disagreement. In cases 1a and 14, we obtained exactly the same numerical changes by both methods. In the rest of the samples, the poor quality of the chromosomes made it difficult to determine the exact karyotype. The presence of markers and multiple subclones may explain the differences between CGH and cytogenetics in these samples. Furthermore, an interphase cytogenetic study with a centromere-specific probe for chromosome 1 was performed in one case with der(1;16)(q10;p10), which leads to partial trisomies of 1q and partial monosomies of 16q. The abnormality was present only in 30% of the cells analysed (Tarkkanen et al, 1993). The low frequency of the clone could explain the normal karyotype found by CGH.

Our CGH analysis shows that many loci frequently show copy number changes in ET. However, the critical and primary event in the tumorigenesis of ET is most likely the t(11;22) or a variant translocation. The secondary abnormalities are the gain of chromosomes 8 and 12, and the gain of DNA sequences in 1q, which agree with cytogenetic studies. As one of the known translocations is a likely primary event, it is possible that these additional changes may have prognostic significance. Owing to the limited number of cases in the present study, testing of the statistical significance of the prognostic effect of these changes is associated with a high risk of a type II statistical error. It is of interest to note, however, that copy number increases in 1q and in chromosomes 8 and 12 were all associated with (non-significant) trends to poor survival. To evaluate this further, a larger number of patients needs to be studied.

### ACKNOWLEDGEMENTS

This study was supported by a grant from the Universitat Autònoma de Barcelona (GA), the Clinical Research Institute of the Helsinki University Central Hospital (MT), the Finnish Medical Society Duodecim (MT), the Foundation of Orthopaedics and Traumatology in Finland (MT), the Finnish Cancer Society (MT, SK) and the Norwegian Cancer Society (AF).

### REFERENCES

Ambros IM, Ambros PF, Strehl S, Kovar H, Gadner H and Salzer-Kuntschik M (1991) MIC2 is a specific marker for Ewing's sarcoma and peripheral primitive neuroectodermal tumors. *Cancer* **67**: 1886–1893

Delattre O, Zucman J, Plougastel B, Desmaze C, Melot T, Peter M, Kovar H, Joubert I, de Jong P, Rouleau G, Aurias A and Thomas G (1992) Gene fusion with an *ETS* DNA-binding domain caused by chromosome translocation in human tumours. *Nature* **359**: 162–165

Douglass EC, Rowe ST, Valentine M, Parham D, Meyer WH and Thompson EI (1990) A second nonrandom translocation, der(16)t(1;16)(q21;q13), in Ewing sarcoma and peripheral neuroectodermal tumor. *Cytogenet Cell Genet* **53**: 87–90

Engelkamp D, Schäfer BW, Mattei MG, Erne P and Heizmann CW (1993) Six S100 genes are clustered on human chromosome 1q21: identification of two genes coding for the two previously unreported calcium-binding proteins S100D and S100E. *Proc Natl Acad Sci USA* **90**: 6547–6551

Forus A, Flørenes VA, Mælandsmo GM, Meltzer PS, Fodstad Ø and Myklebost O (1993) Mapping of amplification units in the q13–14 region of chromosome 12 in human sarcomas: some amplica do not include *MDM2*. *Cell Growth Differ* **4**: 1065–1070

Forus A, Weghuis DO, Smeets D, Fodstad Ø, Myklebost O and van Kessel AG (1995*a*) Comparative genomic hybridization analysis of human sarcomas. II. Identification of novel amplicons at 6p and 17p in osteosarcomas. *Genes Chrom Cancer* **14**: 15–21

Forus A, Weghuis DO, Smeets D, Fodstad Ø, Myklebost O and van Kessel AG (1995*b*) Comparative genomic hybridization analysis of human sarcomas. I. Occurrence of genomic imbalances and identification of a novel major amplicon at 1q21–q22 in soft tissue sarcomas. *Genes Chrom Cancer* **14**: 8–14

Forus A, Weterman MAJ, Van Kessel AG, Berner J-M, Fodstad Ø and Myklebost O (1996) Characterisation of 1q21–22 amplifications in human sarcomas by CGH and molecular analysis. *Cytogenet Cell Genet* **72**: 148

Gibbs S, Fijneman R, Wiegant J, Geurts van Kessel A, van de Putte P and Backendorf C (1993) Molecular characterisation and evolution of the SPRR family of keratinocyte differentiation markers encoding small proline-rich proteins. *Genomics* **16**: 630–637

Hattinger CM, Rumpler S, Strehl S, Ambros IM, Zoubek A, Stark B, Koscielniak E, Gadner H and Ambros PF (1996) Chromosomal aberrations in Ewing tumors: an evaluation by conventional cytogenetics and in situ hybridization techniques. 5th European Workshop on Cytogenetics and Molecular Genetics of Human Solid Tumours, Baveno, Italy, 173.

Hohl D, de Viragh PA, Amiguet-Barras F, Gibbs S, Backendorf C and Huber M (1995) The small proline-rich proteins constitute a multigene family of differentially regulated cornified cell envelope precursor proteins. *J Invest Dermatol* **104**: 902–909

Huang LS, Bock SC, Feinstein SI and Breslow JL (1985) Human apolipoprotein B cDNA clone isolation and demonstration that liver apolipoprotein B mRNA is 22 kilobases in length. *Proc Natl Acad Sci USA* **82**: 6825–6829

Jeon I-S, Davis N, Braun BS, Sublett JE, Roussel MF, Denny CT and Shapiro DN (1995) A variant of Ewing's sarcoma translocation t(7;22) fuses the *EWS* gene to the ETS gene *ETV1*. *Oncogene* **10**: 1229–1234

Kallioniemi A, Kallioniemi O-P, Sudar D, Rutovitz D, Gray JW, Waldman F and Pinkel D (1992) Comparative genomic hybridization for molecular cytogenetic analysis of solid tumors. *Science* **258**: 818–821

Kallioniemi O-P, Kallioniemi A, Piper J, Isola J, Waldman FM, Gray JW and Pinkel D (1994) Optimizing comparative genomic hybridization for analysis of DNA sequence copy number changes in solid tumors. *Genes Chrom Cancer* **10**: 231–243

Ladanyi M, Lewis R, Jhanwar SC, Gerald W, Huvos AG and Healey JH (1995) *MDM2* and *CDK4* gene amplification in Ewing's sarcoma. *J Pathol* **175**: 211–217

McKeon C, Thiele CJ, Ross RA, Kwan M, Triche TJ, Miser JS and Israel MA (1988) Indistinguishable patterns of protooncogene expression in two distinct but closely related tumors: Ewing's sarcoma and neuroepithelioma. *Cancer Res* **48**: 4307–4311

Miller SA, Dykes DD and Polesky HF (1988) A simple salting out procedure for extracting DNAs from human nucleated cells. *Nucleic Acids Res* **16**: 1215

Mitchell EL, White GR, Santibanez-Koref MF, Varley JM and Heighway J (1995) Mapping of gene loci in the q13–15 region of chromosome 12. *Chrom Res* **3**: 261–262

Mitelman F (1994) *Catalog of Chromosome Aberrations in Cancer*, 5th edn. Wiley-Liss: New York

Mugneret F, Lizard S, Aurias A and Turc-Carel C (1988) Chromosomes in Ewing's sarcoma. II. Nonrandom additional changes, trisomy 8 and der(16)t(1;16). *Cancer Genet Cytogenet* **32**: 239–245

Navarro S, Cavazzana AO, Llombart-Bosch A and Triche TJ (1994) Comparison of Ewing's sarcoma of bone and peripheral neuroepithelioma. An immunocytochemical and ultrastructural analysis of two primitive neuroectodermal neoplasms. *Arch Pathol Lab Med* **118**: 608–615

Presland RB, Haydock PV, Fleckman P, Nirunsuksiri W and Dale BA (1992) Characterisation of the human epidermal profilaggrin gene. Genomic organisation and identification of an S-100 like calcium binding domain at the amino terminus. *J Biol Chem* **267**: 23772–23781

© Cancer Research Campaign 1997

Smith SH, Weiss SW, Jankowski SA, Coccia MA and Meltzer PS (1992) SAS amplification in soft tissue sarcomas. *Cancer Res* **52**: 3746–3749

Suijkerbuijk RF, Olde Weghuis DEM, Van Den Berg M, Pedeutour F, Forus A, Myklebost O, Glier C, C T-C and Van Kessel AG (1994) Comparative genomic hybridization as a tool to define two distinct chromosome 12-derived amplification units in well-differentiated liposarcomas. *Genes Chrom Cancer* **9**: 292–295

Szymanska J, Tarkkanen M, Wiklund T, Virolainen M, Blomqvist C, Asko-Seljavaara S, Tukiainen E, Elomaa I and Knuutila S (1996*a*) Gains and losses of DNA sequences in liposarcomas evaluated by comparative genomic hybridization. *Genes Chrom Cancer* **15**: 89–94

Szymanska J, Mandahl N, Mertens F, Tarkkanen M, Karaharju E and Knuutila S (1996*b*) Ring chromosomes in parosteal osteosarcoma contain sequences from 12q13–15. A combined cytogenetic and comparative genomic hybridization study. *Genes Chrom Cancer* **16**: 31–34

Tarkkanen M, Kaipainen A, Karaharju E, Böhling T, Szymanska J, Heliö H, Kivioja A, Elomaa I and Knuutila S (1993) Cytogenetic study of 249 consecutive patients examined for a bone tumor. *Cancer Genet Cytogenet* **68**: 1–21

Tarkkanen M, Karhu R, Kallioniemi A, Elomaa I, Kivioja A, Nevalainen J, Böhling T, Karaharju E, Hyytinen E, Knuutila S and Kallioniemi O-P (1995) Gains and losses of DNA sequences in osteosarcomas by comparative genomic hybridization. *Cancer Res* **55**: 1334–1338

Turc-Carel C, Aurias A, Mugneret F, Lizard S, Sidaner I, Volk C, Thiery JP, Olschwang S, Philip I, Berger MP, Philip T, Lenoir GM and Mazabraud A (1988) Chromosomes in Ewing's sarcoma. I. An evaluation of 85 cases and remarkable consistency of t(11;22)(q24;q12). *Cancer Genet Cytogenet* **32**: 229–238

Wolf M, Aaltonen LA, Szymanska J, Tarkkanen M, Blomqvist C, Berner J-M, Myklebost O and Knuutila S (1997). Complexity of 12q13–22 amplicon in liposarcoma – microsatellite repeat analysis. *Genes Chrom Cancer* **18**: 66–70

Zucman J, Melot T, Desmaze C, Ghysdael J, Plougastel B, Peter M, Zucker JM, Triche TJ, Sheer D, Turc-Carel C, Ambros P, Combaret V, Lenoir G, Aurias A, Thomas G and Delattre O (1993) Combinatorial generation of variable fusion proteins in the Ewing family of tumours. *EMBO J* **12**: 4481–4487

© Cancer Research Campaign 1997